# Leaf mineral nutrient remobilization during leaf senescence and modulation by nutrient deficiency

**DOI:** 10.3389/fpls.2015.00317

**Published:** 2015-05-13

**Authors:** Anne Maillard, Sylvain Diquélou, Vincent Billard, Philippe Laîné, Maria Garnica, Marion Prudent, José-Maria Garcia-Mina, Jean-Claude Yvin, Alain Ourry

**Affiliations:** ^1^UMR 950 Ecophysiologie Végétale, Agronomie et Nutritions NCS, Normandie UniversitéCaen, France; ^2^UMR 950 Ecophysiologie Végétale, Agronomie et Nutritions NCS, UNICAENCaen, France; ^3^UMR 950 Ecophysiologie Végétale, Agronomie et Nutritions NCS, INRACaen, France; ^4^Timac Agro SpainOrcoyen, Spain; ^5^UMR1347 Agroécologie, INRADijon, France; ^6^Groupe Roullier, Centre de Recherche International en Agroscience, CRIAS-TAIDinard, France

**Keywords:** remobilization, ionomic, nutrient deficiencies, senescence, crop species, *Brassica napus*, trees

## Abstract

Higher plants have to cope with fluctuating mineral resource availability. However, strategies such as stimulation of root growth, increased transporter activities, and nutrient storage and remobilization have been mostly studied for only a few macronutrients. Leaves of cultivated crops (*Zea mays, Brassica napus, Pisum sativum, Triticum aestivum, Hordeum vulgare*) and tree species (*Quercus robur, Populus nigra, Alnus glutinosa*) grown under field conditions were harvested regularly during their life span and analyzed to evaluate the net mobilization of 13 nutrients during leaf senescence. While N was remobilized in all plant species with different efficiencies ranging from 40% (maize) to 90% (wheat), other macronutrients (K–P–S–Mg) were mobilized in most species. Ca and Mn, usually considered as having low phloem mobility were remobilized from leaves in wheat and barley. Leaf content of Cu–Mo–Ni–B–Fe–Zn decreased in some species, as a result of remobilization. Overall, wheat, barley and oak appeared to be the most efficient at remobilization while poplar and maize were the least efficient. Further experiments were performed with rapeseed plants subjected to individual nutrient deficiencies. Compared to field conditions, remobilization from leaves was similar (N–S–Cu) or increased by nutrient deficiency (K–P–Mg) while nutrient deficiency had no effect on Mo–Zn–B–Ca–Mn, which seemed to be non-mobile during leaf senescence under field conditions. However, Ca and Mn were largely mobilized from roots (-97 and -86% of their initial root contents, respectively) to shoots. Differences in remobilization between species and between nutrients are then discussed in relation to a range of putative mechanisms.

## Introduction

As sessile organisms, higher plants have to cope with a permanently fluctuating availability of soil nutrients, both in space and time. When facing mineral nutrient deficiency, different plant strategies have evolved to optimize acquisition and use of most macro- and some micronutrients. The first strategy relies on an increase in the expression of genes encoding more or less nutrient-specific root transporters (Amtmann and Armengaud, 2009; Gojon et al., 2009) coupled with the second longer term strategy of increased and oriented exploration of the soil by roots resulting from their growth and increased branching (Gruber et al., 2013; Giehl and von Wirén, 2014; Giehl et al., 2014). Root exudation of organic compounds is the third process by which nutrient mobility and phytoavailability of some nutrients can be increased either directly or indirectly through stimulation of rhizobiont activity (Dakora and Phillips, 2002). However, these three first strategies may not be sufficient to buffer any reduction in soil mineral nutrient availability and hence maintain the plant growth rate under varying conditions. The fourth identified strategy has been less described and relies on the remobilization of short or mid-term storage of macro- and micronutrients within the plant, which may be used to buffer a transient lack of mineral uptake by roots. This strategy occurs during vegetative growth when the availability of nutrients in soil is insufficient, mature leaves become sources to support the growth of news organs for example for N (Malagoli et al., 2005) or S (Abdallah et al., 2010).

However, remobilization processes may also occur during reproductive growth when root activity and nutrient uptake generally decrease while new sinks are emerging (Malagoli et al., 2005). In the latter case, remobilization of nutrients is frequently associated with foliar senescence, which makes nutrients available for younger plant organs and contributes to nutrient use efficiency (Himelblau and Amasino, 2001; Fischer, 2007; Avice and Etienne, 2014). Remobilization requires mostly phloem transport. Macronutrients with the exception of Ca (i.e., N, P, S, K, and Mg) are known to be highly mobile in the phloem, while micronutrients (i.e., Fe, Zn, Cu, Ni, Mo, B, and Cl) with the exception of Mn show at least moderate mobility as reported by White (2012).

Seasonal patterns of macronutrient remobilization have also been reported in woody species. Deciduous trees store nutrients during winter, which are remobilized from the trunk each spring to sustain leaf growth as previously shown for N (Millard and Grelet, 2010). Mature trees rely more on the remobilization of N stores for their growth each spring than do small, juvenile trees (Millard et al., 2006). In evergreen trees, it has been shown that macronutrients such as nitrogen and phosphorus are remobilized from leaves (Cherbuy et al., 2001). This remobilization occurs in summer, after vegetative growth and synchronously with leaf shedding. K remobilization occurs in mid-summer, similar to N and P, and could be attributed to K resorption before leaf shedding or the fulfillment of nutrient demands when soil availability is low (Milla et al., 2005).

General appraisals of the remobilization of most nutrients during plant growth are relatively scarce, being only well-documented for the most abundant nutrients such as N, S, and P; and most of the time they have been studied individually. The simplest estimation of nutrient remobilization can be calculated through the “apparent remobilization” method, which relies on the determination of the amount of total nutrient present in the different plant organs at different times of development as previously used by Hocking and Pate (1977). Isotopic labeling, which allows the determination of nutrient fluxes derived from root uptake and by subtraction the remobilization of unlabeled nutrient between tissues is a more precise method, but it is limited by the availability of suitable isotopes. Using stable isotope labeling, it has been shown that N is remobilized from senescing leaves to expanding leaves at the vegetative stage as well as to seeds during the reproductive stage in *Arabidopsis thaliana* and in *Brassica napus* (Malagoli et al., 2005; Diaz et al., 2008; Masclaux-Daubresse et al., 2010). Contrary to this, sulfur remobilization from leaves to the seeds is considered as a process independent of senescence (Abdallah et al., 2011) mostly because most S storage is under a mineral form (SO^2−^_4_ may account for up to 70% of total S in *B. napus* leaves) (Abdallah et al., 2011). On the other hand, phosphorus remobilization has been less well-described in the literature. In wheat, remobilization of P accounted for 56–63% of the grain P content (Masoni et al., 2007). In P-deficient wheat, around 58–90% of P in the grain could be attributed to retranslocated P (Batten et al., 1986), whereas the proportion was substantially lower (up to 21% P) when the roots were continuously well-supplied with P.

Remobilization of micronutrients from leaves has received much less attention than for macronutrients in crops and in woody species because of low concentrations in tissues and probably the limited use of stable isotope [see Waters et al. (2009) or Hegelund et al. (2012) using stable isotopes of Zn]. Yet crops as basic sources of essential micronutrients do not always contain sufficient amounts of these essential nutrients to meet animal or human dietary requirements (Gupta et al., 2001; Alloway, 2008). Over the past 60 years, micronutrient contents (mostly Fe, Zn, Mg, and Cu) have been reduced in edible products, despite concentrations in soil that have either increased or remained stable (Fan et al., 2008). This has been attributed to varietal selection that aimed to achieve higher yields (Fan et al., 2008; Murphy et al., 2008). Consequently, improving the transfer of such micronutrients into edible parts via remobilization from vegetative tissues could be a way to satisfy micronutrient needs. Remobilization of Fe, Cu, and Zn has been specifically investigated. In wheat, the concentration of Fe and Cu in all plant vegetative organs has been shown to drop over time during grain filling (77 and 40–62%, respectively) due to their remobilization (Garnett and Graham, 2005). Remobilization of Zn from leaves to the grain is substantial in wheat (Kutman et al., 2012) and barley (Hegelund et al., 2012) but affected by Zn availability during post anthesis. Kutman et al. (2011) reported that more Fe (80%) than Zn (50%) in wheat grains was derived from leaf remobilization. Iron and Zn remobilization from wheat leaves has been shown to be impaired in *Ta*NAM (NAC-type transcription factor) RNAi line compare to WT plants and that the extent of net Fe and Zn remobilization was dependent on availability of mineral input in both lines (Waters et al., 2009). This *Ta*NAM RNAi wheat line was then characterized as having a delayed leaf senescence which provided a yield advantage under optimal conditions but with lower grain nutritional quality, resulting from a reduced remobilization of most minerals to the grains (Guttieri et al., 2013). Shi et al. (2012) demonstrated that leaf Fe remobilization with barley senescing leaves was stimulated by N deficiency, that increased phytosiderophore synthesis and hence Fe solubility. However, using dark induced leaf senescence, they suggested that Fe remobilization from mature leaves was independent of N remobilization.

To date, only a few studies have attempted to describe remobilization of all essential nutrients during plant senescence (Hocking and Pate, 1977; Himelblau and Amasino, 2001; Waters and Grusak, 2008; Moreira, 2009). Hocking and Pate (1977) using three legume species (*Pisum sativum, Lupinus albus, Lupinus augustifolius*) grown under controlled conditions, described three classes of nutrients with diverging leaf remobilization efficiencies: N, P, and K (60–90% of remobilization from leaves); Mg, Zn, Mn, Fe, and Cu (20–60%); and Na and Ca (<20%). In *Musa spp*, N, P, K, Mg, and Cu had a high remobilization rate compared to other nutrients investigated. B, Zn, and S had an intermediate rate whereas Ca, Fe, and Mn had low remobilization to the fruits (Moreira, 2009). In *A. thaliana*, the levels of Cu, Fe, K, Mo, N, P, S, and Zn in leaves drop by <40% during senescence suggesting that these nutrients are mobilized from senescing leaves (Himelblau and Amasino, 2001). Waters and Grusak (2008) using the same species but with different ecotypes showed that remobilization of some nutrients was genetically influenced (Cu, Zn, or S) or kept under the same magnitude whatever the ecotype (K for example). On average, they considered that remobilization of K accounted for about 48% of seed contents, only for 6–30% of Fe, P, S, Zn, and Cu, while Ca, Mg, or Mn were not remobilized at all. Moreover, it was shown that the *ysl1ysl3* mutant impaired in two metal-chelate transporter genes had impaired movement of Cu and Zn from the senescing rosette leaves.

Therefore, the aims of the current study were firstly to determine whether the different plant species remobilize minerals differently. In order to do so, the apparent remobilization was quantified during the leaf senescence in eight plant species. They included five crop species (*Triticum aestivum, Hordeum vulgare, Brassica napus, Pisum sativum, Zea mays*) and three woody species (*Quercus robur, Alnus glutinosa, Populus nigra*) grown in field conditions. This choice of plant species was dependent on their economic importance, their ability to fix atmospheric N (*Pisum sativum* and *Alnus glutinosa*), their photosynthetic system (*Zea mays* as a C4) and their different patterns of growth and senescence (*Quercus robur* vs. *Populus nigra*). Secondly, the putative remobilization of each nutrient was quantified from mature leaves (i.e., before senescence) and from roots using *B. napus* grown in hydroponic culture and subjected to 13 individual nutrient deficiencies in order to determine if remobilization is induced or not by nutrient deficiency independently of senescence.

## Materials and methods

### Harvesting leaves during senescence

Eight species (*Brassica napus* L., *Triticum aestivum* L., *Hordeum vulgare* L., *Zea mays* L., *Pisum sativum* L, *Quercus robur* L., *Alnus glutinosa* L. Gaertn, and *Populus nigra* L.) grown under field conditions or on soil under greenhouse conditions (*Pisum sativum* L.) were chosen in order to perform regular leaf harvests from early stages of development up to leaf fall. Leaves from three woody species (*Q. robur, A. glutinosa, P. nigra*) were harvested from the lower canopy (below 4 m height) with three replicates, each of them consisting of at least 50 leaves from three different trees. *Q. robur* and *P. nigra* were harvested from trees located in the edges of the same grassland and therefore under the same soil (analysis is given in SD2) and climatic conditions. The age of the different *Q. robur, A. glutinosa*, and *P. nigra* trees was calculated using the trunk diameter at breast height (Claessens et al., 2010; Rohner et al., 2013) and was estimated to be between 96 and 134, 40 and 62, and 31 and 39 years, respectively. Only *Q. robur* trees were under reproductive phase. It was assumed that leaf development and senescence was synchronous in trees even if the senescence of some leaves may be affected by shading. For annual species, a specific leaf rank was selected. Leaves of four annual species were harvested from field grown plants with three replicates, each of them consisting of at least 15 (*Z. mays*, leaf rank just below the highest ear), 50 (*T. aestivum*, flag leaf), 50 (*H. vulgare*, flag leaf), or 10 leaves [*B. napus*, leaf rank number 20, identified from scars and harvested previously by Malagoli et al. (2005)]. *Z. mays, B. napus, T. aestivum*, and *H. vulgare* were harvested in nearby fields with similar soil (analysis given in SD2) and under the same climatic conditions. *P. sativum* L. cv Cameor were grown in controlled greenhouse conditions with temperatures of 18°C during the day and 14°C during the night, a 16 h photoperiod and a mean photosynthetically active radiation of 180 μE.m^−2^.s^−1^ guaranteed by the use of high-pressure sodium lamps (MACS 400W; Mazda; Dijon; France) to compensate when daylight was declining. Seeds were sown in 4 L pots filled with a mixture of vermiculite: sand (v:v, 3:1), at a rate of five seeds per pot. Five days after sowing, two plants were removed in order to keep the three most homogeneous ones in the pot. Plants were watered throughout the experiment with a nutrient solution composed of 2 mM NO_3_, 6 mM Ca(NO_3_)_2_ + 4H_2_O, 2.5 mM NaNO_3_, 2.4 mM K_2_HPO_4_, 2.4 mM MgSO_4_ + 7H_2_O, 30 μM H_3_Bo_3_, 10.6 μM MnSO_4_ + 7H_2_O, 0.7 μM ZnSO_4_ + 7H_2_O, 3.2 μM CuSO_4_ + 5H_2_O, 1 μM Na_2_MoO_4_ + 2H_2_O, 84 nM CoCl_2_ + 6H_2_O and 50 μM Fe(III)-EDTA. Harvests were all carried out 4 h after the beginning of the photoperiod, and were undertaken on the fifth leaf of the plant, at 10 time points from its emergence until its senescence (period of 69 days). At each time point, three pools of plants were harvested and each pool was composed of two to 20 plants depending on leaf biomass.

**Figure 1:**
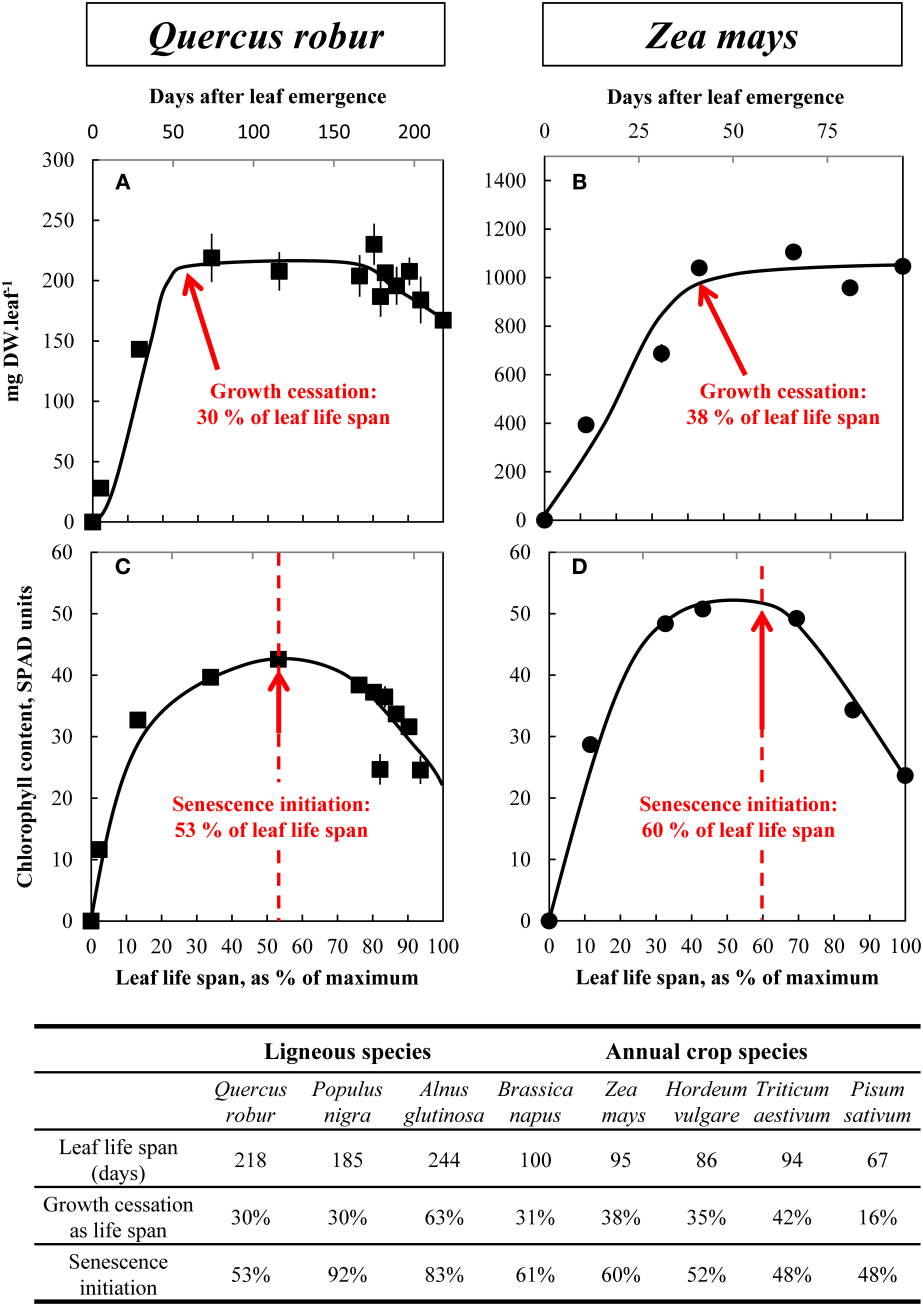
**Changes in whole leaf-blade biomass (A,B) and chlorophyll content (C,D) during the leaf life span (days after leaf emergence) of *Q. robur* (A,C) and *Z. mays* (B,D)**. Dashed lines in **(C)** and **(D)** indicate the beginning of chlorophyll degradation and hence senescence initiation. Vertical bars indicate ± S.E. for *n* = 3 when larger than the symbol. Total leaf life span (days), growth cessation (as % of total leaf life span) and senescence initiation (as % of total leaf life span) are then provided for the eight ligneous and annual crop species. The relative leaf life span (% of maximum) is given on the bottom of each graph.

For each leaf sample, the total number of leaves, fresh weight, and chlorophyll content (SPAD-502 model, Minolta, Tokyo, Japan) were recorded. Dry weight was obtained after placing the samples at 60°C for 4 days (80°C for 48 h for *P. sativum*), and dry samples were kept for further analysis. The life spans of leaves were then estimated by polynomial regressions of leaf dry weight in order to calculate the date of leaf appearance, and hence the total life span of leaves (Figure 1).

### Deficiencies under hydroponic conditions and plant harvests

Seeds of *B. napus* var. Bohème were surface-sterilized by exposure to 80% ethanol for 30 s followed by 20% sodium hypochlorite for 10 min. After 10 washes with demineralized water, seeds were germinated on perlite over demineralized water for 2 days in the dark and then for 1 week under natural light in a greenhouse. Just after first leaf emergence, seedlings were transferred to a 20 L tank (18 seedlings per 20 L-plastic tank) containing the following nutrient solution: 1.25 mM KNO_3_, 1.25 mM Ca(NO_3_)_2_, 0.25 mM KH_2_PO_4_, 0.5 mM MgSO_4_, 0.2 mM EDTA 2NaFe, 10 μM H_3_BO_3_, 5 μM MnSO_4_, 3 μM ZnSO_4_, 0.7 μM CuSO_4_, 0.7 μM (NH_4_)_6_Mo_7_O_24_, 0.1 μM CoCl_2_, 0.04 μM NiCl_2_, 0.1 mM SiO_2_, 1.25 mM CaCl_2_, 0.25 mM KCl. The nutrient solution was buffered to pH 6.5 with 2 mM CaCO_3_ and was renewed every 2 days. Plants were grown under greenhouse conditions, with a thermoperiod of 20/17°C day/night and a photoperiod of 16 h. Natural light was supplemented with high-pressure sodium lamps (Philips, MASTER Green Power T400W, Amsterdam, Netherlands) supplying an average photosynthetically active radiation (PAR) of 350 μmol photons m^−2^.s^−1^ at canopy height.

After 20 days of growth, plants were separated into fourteen sets: plants were grown with the complete nutrient solution previously described (control plants) or with nutrient solutions (Supplemental Data SD1) deprived of N, K, P, S, Mg, Ca, Fe, Zn, Cu, Mn, Mo, B, or Ni. The composition of these nutrient solutions was chosen in order to maintain the same concentration of each nutrient. For example, in the P-starvation treatment, 0.25 mM KH_2_PO_4_ was omitted and the K concentration was maintained at 1.75 mM by the addition of KOH.

Four independent samples each consisting of three individual plants were harvested at the beginning of nutrient-depletion (*t*_0_) and after growth cessation (*t*_f_). Growth cessation for each deprivation treatment was detected by non-destructive estimation of plant weight obtained by daily measurements of total plant fresh weight of control and deficient plants. Leaves and petioles present at the beginning of nutrient-depletion (referred as “mature leaves” and “mature petioles,” respectively) were distinguished from leaves appearing during depletion (referred as “young leaves” and “young petioles,” respectively). Correction fluid was used to mark mature leaves and petioles at t_0_ in order to distinguish them from their younger counterparts during later harvests. At each date of harvest (*t*_0_ and *t*_f_), whole roots from control and depleted plants were collected. An aliquot of each tissue was weighed and dried in an oven (60°C) for dry weight (DW) determination and kept for further analysis. Every 5 days throughout the experiment non-destructive determination of chlorophyll content in young and in mature leaves was performed using a SPAD chlorophyll meter (SPAD-502 model, Minolta, Tokyo, Japan). The determination was carried using three replicates of 10 measurements performed on independent leaves.

### Element analysis by Mass Spectrometry

Plant dry samples were ground to a fine powder with inox beads in an oscillating grinder (Mixer Mill MM400; Retsch, Haan, Germany) for further IRMS, ICP-OES and ICP-MS HR analysis. For the analysis of total N and S contents, an aliquot of around 4 mg DW of each plant organ sample was placed in tin capsules for total N and S analysis in order to analyze between 60 and 80 μg N. The total N amount and S amount in plant samples were determined with a continuous flow isotope mass spectrometer (Nu Instruments, Wrexham, United Kingdom) linked to a C/N/S analyser (EA3000, Euro Vector, Milan, Italy). The total N or S amount (*N*_tot_ or *S*_tot_) in a tissue “*i*” at a given time “*t*” was calculated as:

(1) $$N_{tot}\;\left(or\;S_{tot}\right)=\frac{\%\;N_{i,t}\left(or\;S_{i,t}\right)\times DW_{i,t}}{100}$$

K, Ca, Mg, Fe, Zn, Cu, Mo, Mn and B in greenhouse grown samples and in four species (*Q. robur* L.*, A. glutinosa* (L.) Gaertn, *P. nigra* L.*, Z. mays* L.) were analyzed by Inductively Coupled Plasma Optical Emission Spectrometry (ICP-OES, Thermo Scientific iCAP 6500) with prior microwave acid sample digestion in an Ethos One microwave (Milestone srl, Milano, Italia) (8 mL of concentrated HNO_3_ and 2 mL of H_2_O_2_ for 0.5 g DW). For the quantification by ICP-OES, all the samples were spiked with an internal-standard solution of yttrium to 10 ppm, diluted to 50 ml with Milli-Q water to obtain solutions containing 2.0% (v/v) of nitric acid.

Other nutrients (P and Ni) in greenhouse grown samples and all nutrients in four species (*T. aestivum* L., *H. vulgare* L., *B. napus*, and *P. sativum* L.) were quantified by Inductively High Resolution Coupled Plasma Mass Spectrometry (HR ICP-MS, Thermo Scientific, Element 2^TM^) with prior microwave acid sample digestion (Multiwave ECO, Anton Paar, les Ulis, France) (800 μL of concentrated HNO_3_, 200 μL of H_2_O_2_ and 1 mL of Milli-Q water for 40 mg DW). For the determination by HR ICP-MS, all the samples were spiked with two internal-standard solutions, gallium and rhodium, respectively, for a final concentration of 10 and 2 μg.L^−1^, diluted to 50 ml with Milli-Q water to obtain solutions containing 2.0% (v/v) of nitric acid, then filtered at 40 μm using a teflon filtration system (Filtermate, Courtage Analyses Services, Mont-Saint-Aignan, France). Quantification of each element was performed using external standard calibration curves. The amount of element in each tissue was then calculated as previously explained for N and S.

### Calculations and statistical analysis

Data obtained under field conditions relied on three independent biological replicates each corresponding to at least three individual plants while data obtained for plants grown in the greenhouse relied on four independent biological replicates, each corresponding to a pool of four plants. Data are given in the text as the mean ± S.E. For greenhouse experiments, data were checked for normality (Shapiro test) and accordingly, were compared using the parametric test of Student. If the normality condition was not reached, the non-parametric Wilcoxon's test was used. Significance of difference are explained in the legend of each figure (^*^P-values < 0.05; ^**^P-values < 0.01; ^***^P-values < 0.001. All tests were performed using R software (http://www.r-project.org).

The daily apparent nutrient remobilization (ANR) of nutrient from leaves harvested from field conditions was calculated as the slope (–*a* which corresponds to nutrient decrease in μg.leaf^−1^.day^−1^) of the linear regression between one nutrient i amount per leaf as a function of time using the following formula:

(2) $$AN_{i}\left(\text{t}\right)=AN_{\text{i}\left(\text{max}\right)}-a\;\times \;time_{\left(days\right)}$$

where *AN*_i(max)_ is the maximum amount of nutrient *i* during leaf life span and *AN*_i_(*t*) is the amount of nutrient *i* at time *t*.

Data points (*n* = 12 at least for *Z. mays* and up to *n* = 42 for *A. glutinosa*) used for such linear correlation were those located between the harvest showing the highest amount of nutrient i in leaf [*AN*_i(max)_], up to the harvest preceding leaf fall. The significance of remobilization of a nutrient i was evaluated by an ANOVA test of the correlation coefficient of the linear regression. The confidence interval for the slope (-*a*) was then calculated using a Student test. The total apparent remobilization of nutrient *i* in leaf was then calculated as:

(3) $$\text{ANR}\left(\%\right)=\frac{-\text{a}\times \text{D}}{{\text{AN}}_{\text{i}(\text{max})}}$$

where *D* is the duration i.e., the number of days during which the nutrient i amount decreased.

For plants grown with nutrient deprivation under hydroponic conditions, the calculation of ANR (%) was done using the following formula:

(4) $$ANR\left(\%\right)=\frac{AN_{i\left(t0\right)}-AN_{i(tf)}}{AN_{i(t0)}}\times 100$$

where AN_i(t0)_ and AN_i(tf)_ are the amount of nutrient i in one tissue before the nutrient deficiency and after the growth cessation, respectively.

Calculations were performed for roots, mature leaves and mature petioles. Moreover, if the total nutrient per plant between *t*_0_ and *t*_f_ was not significantly different, it can be assumed that no nutrient i uptake occurred. In such cases the net flow of nutrient i was then calculated for each tissue and revealed a remobilization from (decreased content: source behavior) or to (increased content: sink behavior) a given tissue. Alternatively, if an increase in the nutrient i content per plant was significant during its deprivation, it follows that traces of nutrient i in the nutrient solution were taken up. In such cases, a minimum (underestimated as some uptake having occurred in the meantime) apparent nutrient remobilization was calculated if a significant decrease in the amount of nutrient i, occurred between times *t*_0_ and *t*_f_ in a given tissue.

## Results

### Changes in leaf dry weight and chlorophyll content in plants grown under field conditions

Figure 1 shows an example of changes in leaf dry weight (DW) (Figures 1A,B) and chlorophyll contents (Figures 1C,D) as a function of leaf life span, for *Q. robur* and *Z. mays* (for other species see Supplemental Data SD3–10, A and B). The maximum leaf DW was measured 65 and 36 days after leaf emergence, respectively, which correspond to 30 and 38% of the leaf total life span. Leaf life span was shorter for annual species (100, 95, 86, 94, and 67 days, respectively for *B. napus, Z. mays, H. vulgare, T. aestivum*, and *P. sativum*) than for tree species (218, 185, and 244 days, respectively for *Q. robur, P. nigra*, and *A. glutinosa*). The cessation of leaf growth occurred after 16 and 63% of the leaf life span for *P. sativum* and *A. glutinosa*, respectively, and occurred at around 30–40% of the leaf life span for all other species. Leaf DW remained almost the same and was correlated with leaf area (data not shown), except for *T. aestivum, H*. *vulgare*, and *B. napus* for which a significant decrease in leaf DW was recorded during the last 5 weeks in *T. aestivum* and *H*. *vulgare* and the last 20 days in *B. napus* (by 33.6 ± 0.86, 59.6 ± 0.31, and 27.4 ± 5.51%, respectively, see Supplemental Data SD8, 9, and 10A). To estimate leaf aging, i.e., senescence initiation, chlorophyll accumulation, and its subsequent decrease were estimated. In all species, relative chlorophyll content increased during early growth until it reached a plateau (Figures 1C,D) after 53 and 60% of leaf life span for *Q. robur*, and *Z. mays*, respectively. Then, a significant drop in chlorophyll content (−42.0 ± 2.98% in *Q. robur* and −53.4 ± 0.27 % in *Z. mays*) occurred in leaves revealing senescence initiation. For most species, senescence seemed to start between 48 and 61% of the leaf life span, except in two tree species: *A. glutinosa* (83%) and *P. nigra* (92%). Late senescence of *P. nigra* and *A. glutinosa* was concomitant with the occurrence of the first atmospheric freezing temperature (end of September and end of October, respectively).

### Changes in nutrient contents during leaf life span in plants grown under field conditions and estimated net remobilization

**Figure 2:**
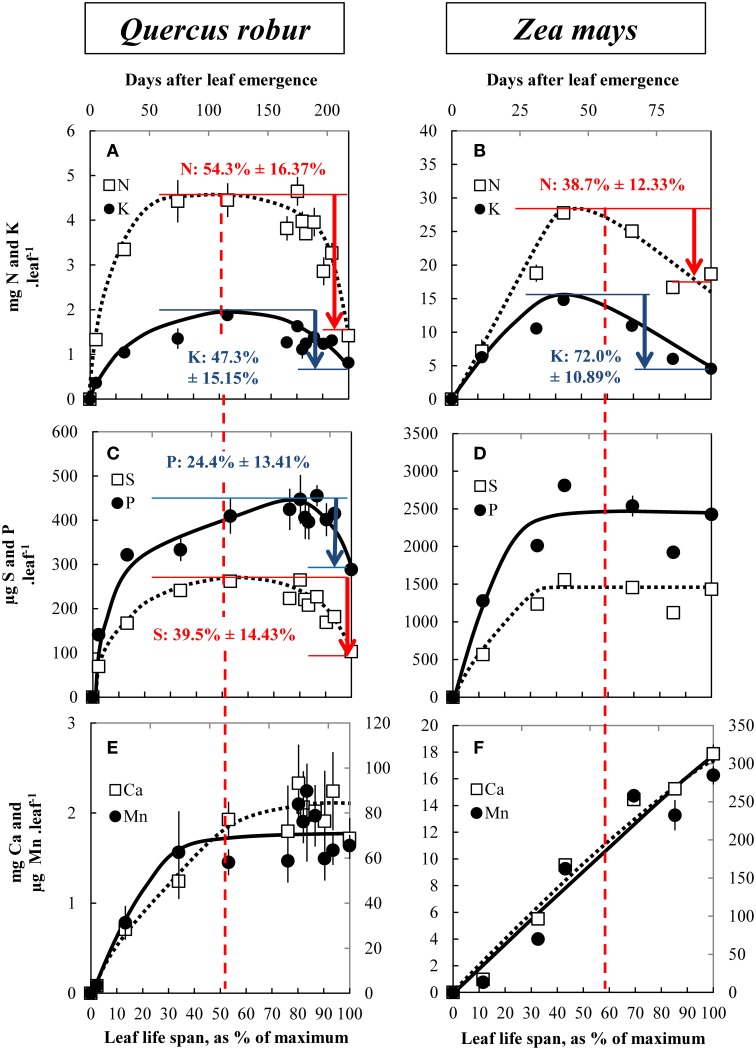
**Changes in nitrogen and potassium (A,B), sulfur and phosphorus (C,D) and calcium and manganese (E,F) contents in leaves during their leaf life span in *Q. robur* (A,C,E) and *Z. mays* (B,D,F)**. Vertical bars indicate ± S.E. for *n* = 3 when larger than the symbol. The apparent nutrient remobilization (ANR) is given with its confidence interval (*p* = 0.05). Dashed lines indicate the beginning of chlorophyll degradation and hence senescence initiation. The relative leaf life span (% of maximum) is given on the bottom of each graph.

As typical examples, Figure 2 presents selected mineral nutrients that follow contrasting but typical behaviors such as N and K (Figures 2A,B), S and P (Figures 2C,D), and Ca and Mn (Figures 2E,F) in *Q. robur* and *Z. Mays*. Other nutrients and other species are also provided as Supplemental Data (SD3–10). All nutrients with the exception of Ca and Mn were accumulated up to maximum DW accumulation or slightly latter. At that point their nutrient contents per leaf reached a plateau (except for Mn and Ca in *Z. Mays*, Figure 2F and Ca in *Q. robur*, Figure 2E) and, according to species, decreased. From maximum to minimum content, nitrogen net remobilization can be estimated at 54.3% ± 9.50 in *Q. robur* and at 38.7% ± 6.80 in *Z. mays*. Potassium was remobilized by 47.3% ± 8.87 in *Q. robur* and by 72.0% ± 6.01 in *Z. mays*. For both species, nutrient remobilization rates coincided with senescence (Figures 1C,D) and remobilization was initiated half way through the leaf life span: at 53% and between 50 and 60% of the leaf life span in *Q. robur* and *Z. mays*, respectively. However, discrepancies occurred for given nutrients and according to plant species; for example a net remobilization of S and P was measured in *Q. robur* (by 39.5% ± 8.40 and 24.4% ± 7.81, respectively), both macronutrients that were apparently not remobilized in *Z. mays* (Figure 2D). Other nutrients such as Ca and Mn were not subjected to an apparent remobilization (Figures 2E,F), being constantly accumulated even during senescence (Ca in *Q. robur*, Ca and Mn in *Z. mays*).

**Figure 3:**
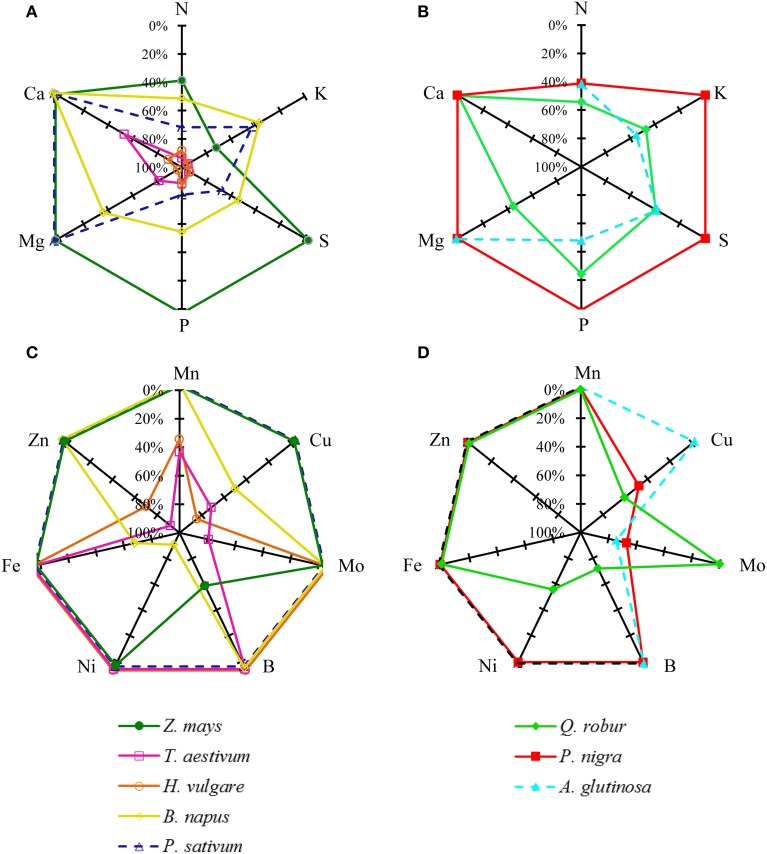
**Apparent nutrient remobilization (ANR) of macronutrients (A,B): N, K, P, S, Ca, Mg, and micronutrients (C,D): Zn, Fe, Mn, B, Ni, Cu, and Mo, expressed as % of maximum nutrient content during leaf senescence in three tree species; *Q. robur, P. nigra, A. glutinosa* and five crop species: *Z. mays, T. aestivum, H. vulgare, P. sativum* and *B. napus***. Species grown on the same soil are represented by a solid line and species like *A. glutinosa* and *P. sativum* harvested on another soil are represented by a dashed line. The ANR was calculated from Equation (3) given in material and methods. Each value is the mean for *n* = 9. For reasons of clarity, confidence intervals are not indicated (see Supplemental Data SD3–10).

Based on similar data collected for the eight plant species (provided as Supplemental Data SD3–10), the apparent nutrient remobilization (ANR) was calculated for all nutrients and all species and is given in Figure 3. Interpretation of these results can be made at two levels, species and nutrients. N was always remobilized, whatever the plant species, with a minimum rate of remobilization of −38.7%. Nitrogen remobilization coincided with senescence as illustrated by chlorophyll content (Supplementary Data SD3–10B). The N remobilization efficiency varied with species. N was remobilized by about −90% in *T. aestivum* and *H. vulgare*, by about −72% in *P. sativum*, by −50% in *Q. robur*, and only by −40% in *Z. mays*, in *P. nigra* and *A. glutinosa*. Remobilization rates of K, S, P, and Mg were highly variable depending on the plant species. Neither P nor S were remobilized in *Z. mays* and *P. nigra*, and this was also the case for K in the latter species. Mg was remobilized only in *Q. robur, B. napus, H. vulgare*, and *T. aestivum*. Ca was not remobilized in any of the studied species except for two of the cereals: *H. vulgare* and *T. aestivum*.

Micronutrients were remobilized with various degrees of efficiency. The rate of remobilization varied widely according to species. For example, Fe was remobilized solely in *B. napus*. In contrast, Cu was widely remobilized in *T. aestivum, H. vulgare, B. napus, P. nigra*, and *Q. robur*.

Taking these results together, macronutrient apparent remobilization efficiency (Figure 3A) could be evaluated for each species. Firstly, considering crop species grown on the same soil, a first group including *T. aestivum* and *H. vulgare* remobilized all macronutrients very efficiently at a rate close to −80%. A second group of plant species with an intermediate apparent remobilization efficiency for macronutrients is characterized by *B. napus* while *Z. mays* corresponds to the third group characterized by a low macronutrient net remobilization efficiency. Grown under different conditions, others species match with the second group (*P. sativum, Q. robur* and *A. glutinosa)* or the third group (*P. nigra*). The same species typology was found for remobilization of micronutrients such as Mn and Zn (Figure 3B). Remobilization of Fe, B, Ni, Cu, Mo, and Mn remained more variable between plant species.

### Alteration of *B. napus* growth during mineral deprivation or deficiency

**Figure 4:**
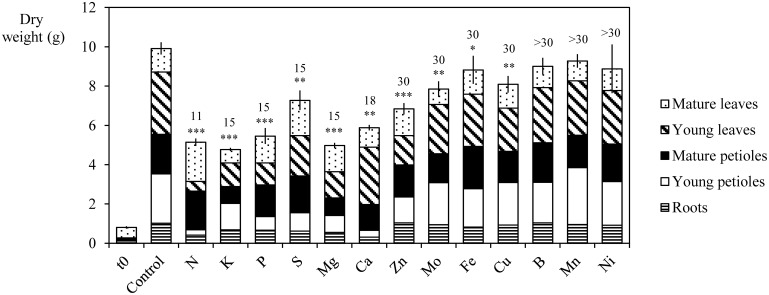
**Dry weight of young petioles, young leaves, mature petioles, mature leaves, and roots of *Brassica napus* L. at *t* = 0 and after 30 days of culture in control plants and in N, P, S, K, Mg, Ca, Zn, Mo, Cu, Ni, Fe, B, or Mn deficient plants**. The number of days required to reach a significant growth reduction is given for each deficiency on the top of each column. Significant differences in total plant dry weight between deficient and control plant are indicated by ^*^, ^**^, or ^***^, for *P* < 0.05, *P* < 0.01, or *P* < 0.001, respectively. Vertical bars indicate S.E. for *n* = 4.

Figure 4 synthesizes the influence of nutrient deprivation or deficiency on the growth of *B. napus* L. whole plants. During the experiment, whole plant biomass progressively increased for all treatments despite nutrient deficiency. After 30 days of culture, the whole plant DW of control plants increased from 0.8 ± 0.06 to 9.9 ± 0.31 g. Biomass production was not significantly different between Ni, B, and Mn deficient and control plants despite a significant reduction of chlorophyll content under B and Mn deficiency (unshown data). The largest reductions involved N-, K-, Mg-, and P-deprived plants whose total DWs were decreased by 48.0 ± 1.24, 51.8 ± 0.81, 49.7 ± 0.98, and 44.9 ± 2.28%, respectively, relative to control plants. Growth cessation was observed after 11 days for plants with N deficiency treatment and 15 days for K, Mg, P, and S-deprivation. This was mostly the result of a significant reduction in the growth of roots and mature and young petioles, while the DW production by mature leaves was not significantly reduced. Other nutrient deficiencies such as -S, -Ca, -Zn, -Mo, -Cu, and -Fe treatments also induced a significant reduction in total plant DW. These were greater for Ca, Zn, and S deficiencies (by 42.6 ± 1.57, by 30.8 ± 1.79, and by 26.5 ± 2.82%, respectively) than for Cu, Mo, and Fe deficiencies (by 18.3 ± 2.53, by 20.7 ± 2.37, and by 10.8 ± 3.97%, respectively).

### Apparent nutrient remobilization in *B. napus* during mineral deprivation or deficiency

**Figure 5:**
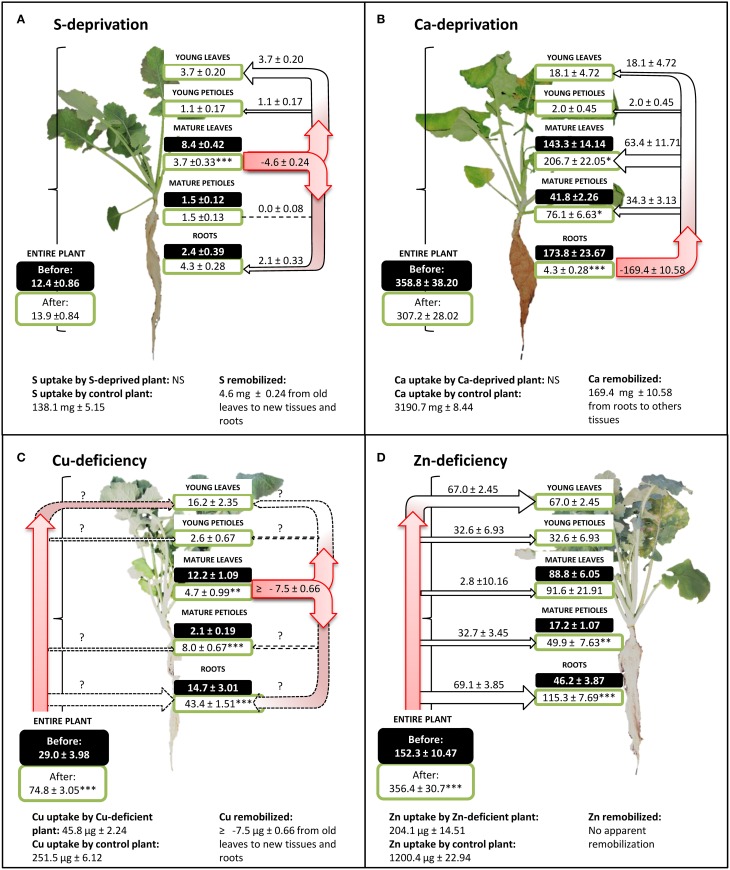
**Sulfur (A), calcium (B), copper (C), and zinc (D) contents per plant in hydroponically grown *Brassica napus* subjected to S, Ca, Cu, and Zn deficiency, respectively**. For each nutrient, uptake by control plants (unlimited supply of nutrients) is given as the mean ± S.E. for *n* = 4. Black and white boxes indicate nutrient content for each organ, before and after nutrient deficiency, respectively, and are given as the mean ± S.E. for *n* = 4. Apparent nutrient remobilization (negative value) from an organ was calculated from Equation (4) given in Material and Methods and allocation (positive value) as well as allocation of nutrient taken up by roots are given as the mean ± S.E. for *n* = 16. Level of significance are indicated by ^*^, ^**^, or ^***^, for *P* < 0.05, *P* < 0.01, or *P* < 0.001, between nutrient deficient plants and *t* = 0 control plants. Question marks indicate flows that cannot be calculated. NS: No significant uptake.

Figure 5 provides four examples (S, Ca, Cu, and Zn) of nutrient uptake, apparent remobilization and further allocation within the plant in control and nutrient deprived plants. The simplest case was found for S (Figure 5A), for which no change in plant S content occurred during deficiency. Plant S content was not significantly different at the beginning and at the end of S-deficiency, being 12.4 ± 0.86 and 13.9 ± 0.84 mg S.plant^−1^, respectively, revealing a lack of significant S uptake from potential trace amounts in the nutrient solution. In the meantime, S uptake by control plants was 138.1 ± 5.15 mg S.plant^−1^. However, sulfur distribution within plant tissues was strongly modified during S deficiency, increasing in roots (+2.1 ± 0.33 mg S), young leaves (+3.7 ± 0.20 mg S), and petioles (+1.1 ± 0.17 mg S) at the expense of mature leaves whose S content decreased by −4.6 ± 0.24 mg S (from 8.4 ± 0.42 to 3.7 ± 0.33 mg S.plant^−1^). In this case, the net remobilization of S can be calculated as −55.2 ± 2.10%.

The same calculations can be done for Ca (Figure 5B) and the conclusions were similar for Mn. During Ca-deprivation, no significant Ca uptake occurred but Ca distribution was also widely affected by deficiency. Root Ca content dropped during deficiency by −169.5 ± 10.58 mg Ca (from 173.8 ± 23.67 to 4.3 ± 0.28 mg Ca) indicating a massive net remobilization of −97.4 ± 0.16% of root Ca to all shoot tissues.

The third type of response concerns Cu. During Cu-deficiency, Cu content per plant rose from 29.0 ± 4.0 to 74.8 ± 3 μg (Figure 5C) while control plants accumulated 251.5 ± 6.12 μg of Cu in the same period. The slight increase in total Cu in Cu-depleted plants was the result of Cu traces found in the mineralized water used for the nutrient solution (0.47 ± 0.00 μM), revealing a situation of deficiency rather than deprivation. However, Cu content in mature leaves of Cu-depleted plants was reduced from 12.2 ± 1.09 to 4.7 ± 0.99 μg Cu indicating a remobilization of −61.4 ± 4.2% of the Cu initially present in these leaves, which is probably minimized under a depletion situation compared to a deficiency condition.

The fourth type of response involved Zn, Fe, B, and Ni. The limitation of Zn availability (Figure 5D) greatly reduced total Zn uptake by −83.0 ± 0.40% but did not change the Zn partitioning within different plant tissues (Figure 5D). In all organs, the Zn amount increased during Zn-deficiency except in mature leaves where no significant change was observed. There was no apparent remobilization of Zn. Similar to Zn, there was no apparent remobilization of Fe, B, and Ni, but traces of these micronutrients were also found in the nutrient solution.

**Figure 6:**
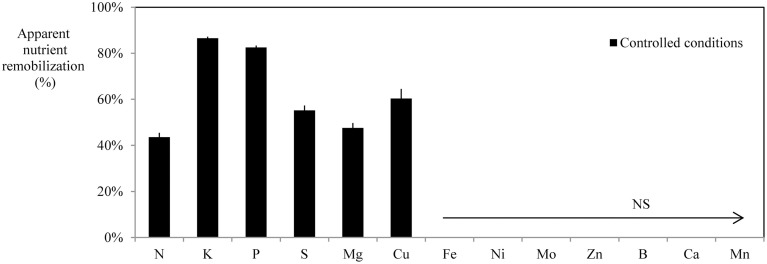
**Apparent nutrient remobilization (ANR), expressed as % of maximum nutrient content in leaves of *B. napus* grown under field condition (calculated from Equation (3) given in material and methods, *n* = 12 or 15 depending on the nutrient) or in mature leaves of hydroponically grown plants [calculated from Equation (4) given in Material and Methods, *n* = 16] subjected to individual nutrient deficiency**. Vertical bars indicate ± S.E.

Figure 6 indicates the range of net nutrient remobilizations from leaves under deficiency or deprivation in controlled hydroponic conditions. *B. napus* remobilized N, P, K S, Mg, and Cu from leaves under deficiencies. Even if the nutritional conditions and the developmental stage of *B. napus* under field conditions were different (Figure 3), it must be pointed on that N, P, K, S, Mg, and Cu were also remobilized from leaves.

## Discussion

### General patterns of nutrient accumulation and remobilization during leaf development

The first results of this study synthetized in the Figure 3 show that mineral nutrients have different patterns of leaf accumulation and remobilization, and moreover that remobilization efficiencies are affected by plant species. During the leaf life span, nutrients were accumulated up to a maximum level corresponding to the period of maximum leaf DW or slightly latter. After this point the content per leaf reached a plateau and, according to species, decreased or continued to accumulate. Amongst the nutrients, three typical patterns of remobilization during leaf senescence can be described (Figure 2).

The first pattern is characterized by a remobilization that occurred at the start of leaf senescence, as revealed by chlorophyll degradation (Figures 1C,D) whatever the plant species. This is the case for N (Figures 2A,B) whose main storage forms are mainly organic, such as proteins and amino acids. However, the efficiency of leaf N remobilization was highly variable as previously reported in the literature for species such as *B. napus* according to leaf ranks (Malagoli et al., 2005) or to N availability (Desclos et al., 2009) or *A. thaliana* (Himelblau and Amasino, 2001).

The second pattern of nutrient accumulation and remobilization encompasses phloem-immobile nutrients represented by Ca, and Mn (Figures 2E,F). Ca per leaf reached a plateau in *Q. robur, P. sativum*, and *B. napus* but was constantly accumulated throughout the leaf lifetime in *P. nigra, A. glutinosa*, and *Z. mays* (see Supplemental Data SD3–10, G) despite cessation of leaf DW accumulation. This latter pattern is consistent with the general assumption that Ca shows a low phloem mobility (Biddulph et al., 1959; White, 2012). Surprisingly, despite this, Ca appeared to be remobilized from senescing leaves of *H. vulgare* and *T. aestivum* (Figure 3A). This follows findings that, under specific induction conditions, leaf Ca remobilization may occur, as reported for few plant species such as *Glycine max* (Mauk and Noodén, 1992), *Phaseolus vulgaris* (Biddulph et al., 1959) after diethyl ether, NaCl or triiodobenzoic acid applies and *Lycopersicum esculentum* being locally scorched (Malone et al., 2002). Such disturbances induced Ca movement in the xylem by a rapid release of water during the dehydration of the plants (Dayod et al., 2010). Alternatively, a second hypothesis to propose is that these two species have a more effective senescence with a more rapid dehydration characterized by a decrease in biomass and a complete loss of chlorophyll (Supplemental Data SD3–10B). A similar pattern of remobilization was recorded for Mn that was not subjected to an apparent remobilization in all studied plant species except again in *T. aestivum* and in *H. vulgare*. Indeed, like Ca, Mn can be leached out of leaves by water (Nable and Loneragan, 1984). Finally, supplementary results found with *B. napus* grown with Ca or Mn deficiency (Figure 5B for Ca, supplemental data SD8 for Mn) also showed an alternative recycling for these two nutrients, with a massive remobilization (by −97.4% ± 4.63 and −86.1% ± 0.62 of root content, respectively) from roots to the shoots that was probably via xylem transport.

A third pattern of remobilization can be identified for K, P, S, and Mg and is characterized by highly variable efficiencies of remobilization. Remobilization rates of these four nutrients were different depending on the plant species (Figure 3A). Efficient removal of K and P has been observed from *A. thaliana* leaves (Himelblau and Amasino, 2001; Waters and Grusak, 2008) and also from senescing leaves of deciduous trees (Hagen-Thorn et al., 2006). In contrast, Teija Ruuhola (2011) found that K concentrations increased significantly throughout the entire leaf live span in *Betula pendula* while Harvey and van den Driessche (1999) observed a differential remobilization of P and K from *P. nigra* leaves depending on drought status (Harvey and van den Driessche, 1999; Teija Ruuhola, 2011). It has been strongly suggested that remobilization of P is induced by P availability. S followed the same pattern as P and K and it was not remobilized in either *P. nigra* or *Z. mays* grown under field conditions. Indeed, S-remobilization has been described as a senescence-independent process, being mostly induced by S deficiency (Abdallah et al., 2011). The majority of investigations concerning Mg remobilization suggest that its remobilization is rather moderate. In our study, Mg was remobilized only in *B. napus, H. vulgare*, and *T. aestivum*. It has been found previously that remobilization of Mg in leaves occurred in *T. aestivum* (Hocking, 1994) and *Fraxinus excelsior* (Hagen-Thorn et al., 2006) but not in *Q. robur, Tilia cordata, Betula pendula* (Hagen-Thorn et al., 2006), *Pisum* sp (White, 2012), or *A. thaliana* leaves (Himelblau and Amasino, 2001). The storage of these four nutrients differed greatly from N, as they are mainly stored in vacuoles under inorganic forms (SO^2−^_4_, K^+^, Mg^2+^ and PP, Pi). This would provide additional argument to suggest that their remobilization could be independent from senescence, being more likely induced by deficiency.

Some micronutrients such as B, Ni, Mo, Fe, Cu, and Zn can also be included in this third class. These nutrients were remobilized with various degree of efficiency depending on plant species. According to the literature, the efficiency of remobilization of these six nutrients differs greatly between species but could also be affected by environment, although with contradictory findings at times. For example, B-remobilization is recognized as species-dependent; this nutrient is transported into the phloem vessels in a complexed form with some diols or polyols (Blevins and Lukaszewski, 1998). B is remobilized in sorbitol-rich species transporting C as polyols such as *Prunus amygdalus, P. dulcis, P. persica, Malus domestica* but is immobile in sorbitol-poor species including *Ficus carica, Pistacia vera*, and *Juglans regia* (Brown and Hu, 1996). Our findings show that B was remobilized only in *Q. robur* (−71.9% ± 17.38) and *Z. mays* (−58.8% ± 11.51), although we did not find any information in the literature that these species are sorbitol-rich. In our study, apparent Ni remobilization was observed only in *Q. robur* and *B. napus*. Ni mobility is also recognized as species-dependent. Indeed, this nutrient appeared to be mobilized from senescing *Glycine max* leaves (Cataldo et al., 1978) but not from senescing *A. thaliana* leaves (Himelblau and Amasino, 2001). Neumann and Chamel (1986) also reported that phloem mobility of ^63^Ni was more important in *P. sativum* than in *Pelargonium zonale* L., although this mobility remained low (Neumann and Chamel, 1986). Declines in the level of leaf Mo were observed in *A. glutinosa, P. nigra*, and *T. aestivum* (Figure 3B) but did not appear to be mobilized from *P. sativum, H. vulgare, B. napus*, and *Z. mays* leaves. Mo was described as a highly phloem-mobile nutrient with foliar application of Mo being the usual procedure for alleviating deficiency. However, some inefficiency in the remobilization of Mo through the phloem may be suggested (White, 2012). Symptoms of Fe, Cu, and Zn also appeared in young leaves, although the phloem translocation of these nutrients is possible (White, 2012). Our findings show that Zn was remobilized only in *H. vulgare* and in *T. aestivum* as previously observed (Hocking, 1994; Hegelund et al., 2012; Kutman et al., 2012), although in other studies no evidence of Zn remobilization has been reported in *T. aestivum* (Garnett and Graham, 2005) or *P. sativum* (Sankaran and Grusak, 2014). In our study, Cu was remobilized in *H. vulgare, Q. robur, B. napus, T. aestivum*, and *P. nigra* while Fe was only remobilized in *B. napus*. Some reports in the literature have shown Fe and Cu remobilization during leaf senescence in *Q. robur* (Abadia et al., 1996), in *Pinus sylvestris* (Nieminen and Helmisaari, 1996), in *A. thaliana* (Himelblau and Amasino, 2001; Waters and Grusak, 2008), in *G. max* (Mauk and Noodén, 1992), and in *T. aestivum* (Hocking, 1994; Guttieri et al., 2013). In contrast, Shi et al. (2011) did not find any decrease in leaf Fe and Cu content during autumn before leaf fall in nine deciduous and evergreen species. These authors have put forward several hypotheses to explain these results such as the lack of sink activity, release of bound metal as protein constituents, the short distance of transport and phloem loading, and the presence of low molecular weight N compounds to bind metals. All species studied in the current work went through their reproductive phase with fairly strong sinks except *P. nigra* and *A. glutinosa*. Another plausible explanation for the discrepancy in metal translocation was also proposed by Shi et al. (2011) as they suggest that remobilization observed by some authors might be related to a metal-deficiency (due for example to drought conditions or calcareous soil). Another reason for restricted micronutrient remobilization in plant collected under field condition could be due to a contamination from atmospheric contaminants. In summary, the remobilization of nutrients from this third class seems to be strongly affected by nutrient deficiency and species-specific differences. The absence of significant apparent remobilization might be due to (i) a lack of sink activity (*P. nigra* and *A. glutinosa*), (ii) a lack of mobility of the nutrients, (iii) insufficient storage of the nutrients in senescing organs, and finally (iv) an uptake from the soil solution that prevails over remobilization under sufficient root supply.

### Species dependence of nutrient remobilization efficiency

Differences among species in their ability to remobilize macro and micronutrients from leaves were highlighted in this study, but species comparison can be made only when plant were grown under very similar soil, topographic and climatic conditions, which was the case for *H. vulgare, T. aestivum*, and *Z. mays*. The two first species can be considered as the most efficient because they remobilized eight nutrients with high efficiency amongst the 13 essential nutrients monitored (Figure 3) while *Z. mays* only remobilized N, K, and B. A first hypothesis may rely on the effect of the characteristic source/sink ratio of each species that can be strongly influenced by breeding in particular. *T. aestivum* and *H. vulgare* have been selected mostly for yield improvement via increases in grain production per plant leading to a strong reduction in the source to sink ratio. As a consequence, it can be hypothesized that nutrient remobilization has been positively selected toward higher efficiencies. This can be further supported by the high macronutrient apparent remobilization efficiencies recorded in this study that reached nearly -80% for N, K, S, and Mg (Figure 3A) and nearly −40% or more for Mn and Cu (Figure 3B). Moreover, leaf dry weight of *T. aestivum* and *H. vulgare* tended to decrease during the leaf life span (Supplemental Data SD9A and 10A). Such a reduction in leaf DW has been previously reported and explained by the export of amino acids derived from the breakdown of proteins, and this may represent about 40% of the weight loss (as reviewed by Schnyder, 1993). Additionally, the hydrolysis of water soluble carbohydrates such as polyfructans may account for up to 60% of the drop in DW as reported in *T. aestivum* by Wardlaw and Willenbrink (2000). The effect of breeding on remobilization can also be supported by the fact that compared to old cultivars, the new high yielding varieties of winter wheat have been characterized by a larger loss of dry matter from vegetative organs and this effect has been associated with a more rapid and more complete remobilization of water-soluble carbohydrates from the vegetative parts (Mehrhoff and Kühbauch, 1990). *Z. mays* can be characterized by low apparent remobilization efficiency for macronutrients (Figure 3A) with B as the sole remobilized micronutrient. Yet *Z. mays* as well as *T. aestivum* and *H. vulgare* have also been selected for commercially viable traits but with different objectives, focusing on reduction of the plant development cycle (cold tolerance and greater precocity) under conditions of high soil fertility in order to allow its culture in temperate areas. It may then be hypothesized that such breeding conditions did not increase the nutrient remobilization efficiency from leaves. However, it must be kept in mind that remobilization efficiencies estimated in this study, may not be representative of one species as it has been shown that mineral remobilization can be affected by intra-specific variation as previously shown for cucumber (Waters and Troupe, 2011).

*P. nigra* and *Q. robur*, which showed contrasting remobilization efficiencies (Figure 3), were harvested from the same site, which allows a relative comparison. *Q. robur* can be characterized by an intermediate net remobilization efficiency (between −40 and −60%) for macronutrients with a remobilization of Ni, B, and Cu, while *P. nigra* can be characterized as species with low net remobilization efficiencies of macro and micronutrients (Figure 3). In the latter species, Pottier et al. (2015), found a moderate remobilization of Mg in leaves but without significant reduction in Zn, Mn, Fe, or Cu contents in leaves. While remobilization of macronutrients occurred simultaneously with initiation of senescence in the two species, the duration of leaf senescence differed among the species and this may also be linked to the fact that *Q. robur* produced a large amount of reproductive tissue. Such early senescence, which is probably a genetically governed property (Fracheboud et al., 2009), coupled with development of substantial reproductive sinks would therefore explain the high remobilization efficiency for nutrients found in this species compared to species without reproductive development and temperature induced senescence. A very late senescence occurred in *P. nigra*, probably as a result of the arrival of the first freezing atmospheric temperatures. It has been shown that mature leaves of *Populus tremula* senescing rapidly under oxidative stress induced by low temperatures retained a large proportion of their maximum nutrient content at full senescence, probably because phloem activity declined rapidly (Fracheboud et al., 2009).

Overall, the results from this study and the literature show that remobilization of mineral nutrients is a fairly general but complex mechanism. However, following 13 different nutrients suggests that rather different mechanisms will need to be considered: (i) remobilization from organic storage forms (such as for N) tightly linked to senescence, (ii) mineral storage that requires more or less specific transporters (S, Mg, K, P), (iii) the effect of deficiency that increased remobilization compensating under reduced root uptake, and (iv) restricted transport (Zn and possibly Fe). Additionally, to our knowledge, Mn and Ca remobilization from roots, demonstrated in this work, has never been descripted previously, and will require further investigations. Additionally, using eight plant species also suggests that remobilization efficiency is probably affected by previous plant breeding, the plant development scheme (source sink ratio or environmentally induced senescence), and plant evolution with species-specific transport occurring in some cases (B for example).

## Author contributions

AM, AO, SD, and JY conceived and designed the experiments. AM, SD, AO, and PL performed the field experiments. AM, VB, and MP performed the greenhouse experiments. AM, VB, MG, SD, and AO acquired and analyzed of data. AM, AO, and SD wrote the manuscript. VB, PL, MP, MG, JG, and JY revised the manuscript for important intellectual content.

### Conflict of interest statement

The authors declare that the research was conducted in the absence of any commercial or financial relationships that could be construed as a potential conflict of interest.
